# Host phenology regulates parasite–host demographic cycles and eco‐evolutionary feedbacks

**DOI:** 10.1002/ece3.8658

**Published:** 2022-03-16

**Authors:** Hannelore MacDonald, Dustin Brisson

**Affiliations:** ^1^ 6572 Department of Biology University of Pennsylvania Philadelphia Pennsylvania USA

**Keywords:** consumer‐resource cycling, disease ecology, eco‐evolutionary feedbacks, phenology, virulence evolution

## Abstract

Parasite–host interactions can drive periodic population dynamics when parasites overexploit host populations. The timing of host seasonal activity, or host phenology, determines the frequency and demographic impact of parasite–host interactions, which may govern whether parasites sufficiently overexploit hosts to drive population cycles. We describe a mathematical model of a monocyclic, obligate‐killer parasite system with seasonal host activity to investigate the consequences of host phenology on host–parasite dynamics. The results suggest that parasites can reach the densities necessary to destabilize host dynamics and drive cycling as they adapt, but only in some phenological scenarios such as environments with short seasons and synchronous host emergence. Furthermore, only parasite lineages that are sufficiently adapted to phenological scenarios with short seasons and synchronous host emergence can achieve the densities necessary to overexploit hosts and produce population cycles. Host‐parasite cycles also generate an eco‐evolutionary feedback that slows parasite adaptation to the phenological environment as rare advantageous phenotypes can be driven extinct due to a population bottleneck depending on when they are introduced in the cycle. The results demonstrate that seasonal environments can drive population cycling in a restricted set of phenological patterns and provide further evidence that the rate of adaptive evolution depends on underlying ecological dynamics.

## Graphical abstract

Our study shows that host seasonality can drive multiseason epidemic cycles due to feedback between host demography and parasite fitness. Short seasons and synchronous host emergence support parasite densities high enough to drive cycling dynamics as parasites adapt. Furthermore, cycling dynamics generate an evolutionary feedback that slows parasite adaptation by preventing adaptive parasite mutants from invading when host densities have been driven down by high parasite densities.

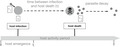

## 1 INTRODUCTION

The impact of interspecies interactions on population demography is a function of both the abundance and activity patterns of the interacting species. For example, the abundance of both a predator and prey species determines the prevalence of predation, which, in turn, alters the demographic dynamics of one or both species. Some ecological interactions have even been shown to drive population sizes to fluctuate cyclically over time (Myers, 2018). Investigations of the ecological interactions leading to population cycles in several predator–prey, herbivore–plant, and parasite–host systems have demonstrated the importance of seasonal activity patterns on interspecies interactions (Abbott & Dwyer, 2007; Greenman et al., 2004; Kamo & Sasaki, 2002; Taylor et al., 2013). Seasonal activity patterns determine the temporal abundance of a population, which modifies the strength of interspecies interactions (Barber et al., 2016; Bewick et al., 2016; Burkett‐Cadena et al., 2011; Miller‐Rushing et al., 2010; Paull & Johnson, 2014). Here, we demonstrate the consequence of seasonal activity patterns on parasite–host population dynamics and how seasonal patterns can result in parasite–host population cycles. Additionally, we explore how parasite–host population cycles can alter the rate of parasite virulence evolution.

Population cycling generally starts with an overexploitation of resources followed by a population crash that allows resources to rebound (Myers & Cory, 2013). In the classic lemming demographic cycles, lemmings overconsume plant resources resulting in dramatic declines in lemming population sizes in subsequent years due to plant scarcity (Krebs, 2013). The plant populations are released from lemming herbivory and increase in abundance, providing sufficient resources for lemming population growth and a restart of the demographic cycle. Intrinsic, delayed density‐dependent drivers such as these can account for the periodic or quasiperiodic oscillatory population dynamics observed in many ecologically coupled systems (Myers, 2018).

Seasonal activity patterns, or phenology, influence the impact of interspecies interactions on demographic dynamics (van Asch & Visser, 2007; Yang & Rudolf, 2010). That is, seasonal activity patterns determine the number and type of interspecies interactions by altering the proportion of a population that is active throughout the year. For example, measles transmission is tightly linked to school terms such that transmission peaks when children are in school and crashes during vacation periods (Fine & Clarkson, 1982; Finkenstädt & Grenfell, 2000). Similarly, variation in demographic dynamics can impact species evolution, for example, resource‐driven changes in host abundance are predicted to impact parasite virulence evolution (Hite & Cressler, 2018). Prior theoretical research demonstrated that the total number of parasite infections, which determines the parasite population size, varied dramatically among different host phenological patterns (MacDonald et al., 2021). Furthermore, the virulence strategies that maximize parasite fitness also differed among phenological patterns due to the differences in the temporal distribution of new infections. However, this work restricted host demographic feedbacks such that the potential for population cycles subsequent effect on evolutionary dynamics could not be investigated.

Changes in the population sizes of interacting species that result from ecological interactions can also influence the rate or direction of evolutionary change (Govaert et al., 2019). These eco‐evolutionary feedbacks arise when evolutionary change occurs on time scales congruent with ecological change. For example, evolutionary adaptation of parasites to a specific host phenological pattern increases parasite densities with a concomitant decrease in host population sizes, which alters both the ecological interactions and the strength and direction of natural selection (MacDonald et al., 2021). Increases in parasite fitness could result in a parasite population that can overexploit hosts leading to temporal oscillations in population sizes with concomitant oscillations in infection prevalence and the strength of natural selection. Thus, host phenology could create conditions that drive the evolution of sufficiently high parasite densities to destabilize host populations and drive population cycles. The resulting population cycles, in turn, could influence the rate and direction of further evolutionary change.

Here, we explore eco‐evolutionary feedbacks driven by parasite infection in a seasonal environment. We extend a previously published modeling framework (MacDonald et al., 2021) to follow within‐season transmission dynamics as well as between‐season parasite and host demography to determine whether evolutionary increases in parasite fitness can lead to cycling population dynamics given different host phenological patterns. Furthermore, we investigate how changes in parasite and host demography, including population cycling, can influence the rate and direction of parasite evolution in seasonal environments. These results contribute to the longstanding goal of revealing how cycling arises by showing how ecological and evolutionary interactions can generate cycling dynamics.

## 2 MODEL DESCRIPTION

### 2.1 Within‐season dynamics

**FIGURE 1:**
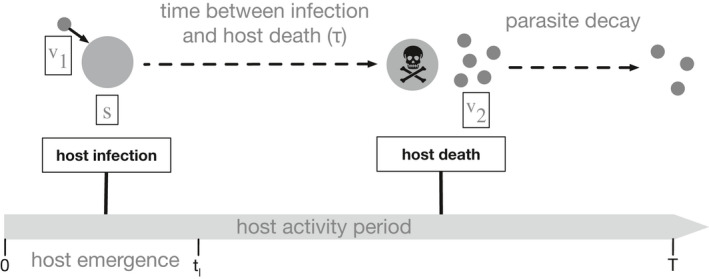
Diagrammatic representation of the infectious cycle within each season. The host population $\left(\hat{s}\left(n\right)\right)$ at the start of season *n* is the offspring of uninfected hosts that survived and reproduced at the end of the prior season. The parasite population at the start of season $n(v_{1}(0)=\hat{v}(n))$ is derived from infected hosts killed by the parasite prior to the end of season *n* − 1 and survived in the environment until the end of the season $\left(v_{2}\left(T\right)\right)$. All parasites emerge at the beginning of the season (t=0) while all hosts emerge at a constant rate between $0\leq t\leq t_{l}$. The rate of new infections is density‐dependent resulting in the majority of infections occurring near the beginning of the season when susceptible host and free parasite densities are high. Parasite‐induced host death at time τ postinfection releases parasite progeny ($v_{2}$) into the environment where they decay in the environment at rate δ. The monocyclic parasite progeny ($v_{2}$) do not infect uninfected hosts within the same season. Parasite progeny that survives in the environment to the end of the season comprises the parasite population that emerges in the following season $\left(v_{2}\left(T\right)=\hat{v}\left(n+1\right)\right)$

The model describes the transmission dynamics of a free‐living, obligate‐killer parasite that infects a seasonally available host (Figure 1). The size of the emerging host cohort in season *n*, $\hat{s}\left(n\right)$, is determined by the number of hosts that reproduced in season *n* − 1. $\hat{s}\left(n\right)$ enters the system at the beginning of the season over a period given by the function *g*(*t*,*t_l_*). Hosts have non‐overlapping generations and are alive for one season. The parasite (*v*) infects hosts and must kill the host to release new infectious progeny. We assume that the parasite is monocyclic and completes one generation per season. The monocyclic constraint is enforced by assuming that only the first generation of parasites in a season, *v*_1_, has enough time to release the second generation of parasites, *v*_2_. This transmission scenario occurs in many natural parasites (e.g., univoltine insects parasitized by ichneumonid wasps (Campbell, 1975; Delucchi, 1982; Kenis & Hilszczanski, 2007)). Parasites may effectively complete only one round of infection per season if the second parasite generation does not have enough time in the season to release new parasites in short‐lived hosts or if the susceptible host stage is present for such a short period of time each season that there are no susceptible host stages available when the first generation of parasites kills infected hosts.

We refer to the generation of parasites that infect the susceptible host stage, *s*, as *v*_1_ and the parasite progeny released from infected hosts as *v*_2_. *τ* is the delay between infection by *v*_1_ and host death when *v*_2_ is released. We ignore the progression of *s* hosts to later life stages as it does not impact transmission dynamics. The initial conditions at the start of each season are $s\left(0\right)=0;\;v_{1}\left(0^{+}\right)=v_{2}\left(0^{‐}\right)=\hat{v}\left(n\right);\;v_{2}\left(\tau \right)=0$, where $\hat{v}\left(n\right)$ is the number of parasites at the beginning of season *n* as determined by the number of parasite progeny produced in *n* − 1. The transmission dynamics in season *n* are given by the following system of delay differential equations:

(1a) $$\frac{ds}{dt}=\hat{s}\left(n\right)g\left(t,t_{l}\right)‐\mu s\left(t\right)‐\alpha s\left(t\right)v_{1}\left(t\right),$$

(1b) $$\frac{dv_{1}}{dt}=‐\delta v_{1}\left(t\right),$$

(1c) $$\frac{dv_{2}}{dt}=\alpha \beta e^{‐\mu \tau }s\left(t‐\tau \right)v_{1}\left(t‐\tau \right)‐\delta v_{2}\left(t\right).$$

where *μ* is the host death rate, *δ* is the decay rate of parasites in the environment, *α* is the transmission rate, *β* is the number of parasites produced upon host death, and *τ* is the delay between host infection and host death (Table 1). We make the common assumption for free‐living parasites that the removal of parasites through transmission (*α*) is negligible (Anderson & May, 1981; Caraco & Wang, 2008; Dwyer, 1994), i.e., (1b) ignores the term $‐\alpha s\left(t\right)v_{1}\left(t\right)$. As virulence is the lifetime reduction in host fitness due to infection, we assume that parasites with shorter times between infection and host death (short incubation periods) are more virulent. Thus, *τ* is equivalent to the inverse of virulence where low virulence parasites have long *τ* and high virulence parasites have short *τ*. All parameters with their respective values are described in Table 1.

**TABLE 1:** Model parameters and their respective values

Parameter	Description	Value
*s*	Susceptible hosts	State variable
*v*_1_	Parasites that infect hosts in current season	State variable
*v*_2_	Parasite progeny released in current season	State variable
$\hat{v}\left(n\right)$	Starting parasite population in season *n*	State variable
$\hat{s}\left(n\right)$	Host cohort in season *n*	State variable
*t_l_*	Length of host emergence period	Time (varies)
*T*	Season length	Time (varies)
*α*	Transmission rate	3.5 × 10^−7^/(parasite × time)
*β*	Number of parasites produced upon host death	200 parasites
*δ*	Parasite decay rate in the environment	2 parasites/parasite/time
*μ*	Host death rate	0.25 hosts/host/time
*τ*	Time between host infection and host death (1/virulence)	Time (evolves)
*σ*	Host fecundity	500 hosts
*ρ*	Density‐dependent parameter	0.0001

The function $g(t,t_{l})$ is a probability density function that captures the per‐capita host emergence rate by specifying the timing and length of host emergence. We use a uniform distribution (U(·)) for analytical tractability, but other distributions can be used.

$$g(t,t_{l})=\left(\begin{array}{cc} \frac{1}{t_{l}} & \;0\leq t\leq t_{l}\\ 0 & \;t_{l}<t\leq T \end{array}\right.$$

where *t_l_* denotes the length of the host emergence period, and *T* denotes the season length. The season begins (*t*_0_ = 0) with the emergence of the susceptible host cohort, $\hat{s}\left(n\right)$. The host cohort emerges from $0\leq t\leq t_{l}$. *v*_2_ parasites remaining in the system at *t* = *T* give rise to the initial parasite population in the following season $\left(v_{2}\left(T\right)=\hat{v}\left(n+1\right)=v_{1}\left(0\right)\right)$. Parasites that have not killed their host by the end of the season do not release progeny. Background mortality arises from predation or some other natural cause. We assume that infected hosts that die from background mortality do not release parasites because the parasites are either consumed or the latency period corresponds to the time necessary to develop viable progeny (Wang, 2006; White, 2011). We solve Equations 1a‐c analytically, Appendix S1.

### 2.2 Between‐season dynamics

We investigate the impact of the feedback between host demography and parasite fitness on parasite evolution by allowing the size of the emerging host cohort be a function of the number of uninfected hosts remaining at the end of the prior season using a difference equation

$$\hat{s}\left(n+1\right)=\frac{\sigma s(T)}{1+\rho s(T)},$$

where *σ* is host reproduction, and *ρ* is the density‐dependent parameter.

In Appendix S1, we find analytical solutions for both $\hat{s}\left(n+1\right)$ and $\hat{v}\left(n+1\right)$. However, we primarily explore the between‐season dynamical behavior of the model numerically as analytical solutions cannot be used in parameter ranges that lead to population cycles. We discuss the stability analysis in more detail in Appendix S1.

### 2.3 Parasite evolution

Evolutionary invasion analysis (Geritz et al., 1998; Metz et al., 1992) was used to study parasite adaptation to different seasonal host activity patterns. We first extend system (1) to follow the invasion dynamics a rare mutant parasite:

(2a) $$\frac{ds}{dt}={\hat{s}}^{*}g\left(t,t_{l}\right)‐\mu s\left(t\right)‐\alpha s\left(t\right)v_{1}\left(t\right)‐\alpha_{m}s\left(t\right)v_{1m}\left(t\right),$$

(2b) $$\frac{dv_{1}}{dt}=‐\delta v_{1}\left(t\right),$$

(2c) $$\frac{dv_{1m}}{dt}=‐\delta_{m}v_{1m}\left(t\right),$$

(2d) $$\frac{dv_{2}}{dt}=\alpha \beta e^{‐\mu \tau }s\left(t‐\tau \right)v_{1}\left(t‐\tau \right)‐\delta v_{2}\left(t\right),$$

(2e) $$\frac{dv_{2m}}{dt}=\alpha_{m}\beta_{m}e^{‐\mu \tau_{m}}s\left(t‐\tau_{m}\right)v_{1m}\left(t‐\tau_{m}\right)‐\delta_{m}v_{2m}\left(t\right).$$

where *m* subscripts refer to the invading mutant parasite and its corresponding traits. The initial conditions at the beginning of each season are $s\left(0\right)=0;\;v_{1}\left(0^{+}\right)=v_{2}\left(0^{‐}\right)={\hat{v}}^{*};\;v_{2}\left(\tau \right)=0;\;v_{1m}\left(0\right)=1;\;v_{2m}\left(\tau \right)=0$, where ${\hat{v}}^{*}$ and ${\hat{s}}^{*}$ are end of season equilibrium densities for parasite and host, respectively. See Appendix S2 for details of the time‐dependent solutions for Equations (2a–2e).

The invasion fitness of a rare mutant parasite depends on the density of $v_{2m}$ produced by the end of the season $\left(v_{2m}\left(T\right)\right)$ in an environment with a resident parasite at equilibrium density ${\hat{v}}^{*}$. When system dynamics are equilibrial, the mutant parasite invades in a given host phenological scenario if the density of $v_{2m}$ produced by time *T* is greater than or equal to the initial $v_{1m}\left(0\right)=1$ introduced at the start of the season $\left(v_{2m}\left(T\right)\geq 1\right)$. When $\tau <T‐t_{l}$, mutant invasion fitness can be found using

(3a) $$v_{2m}\left(T\right)=e^{‐\delta_{m}\left(T‐t_{l}‐\tau_{m}\right)}\left(v_{2m}\left(t_{l}\right)+\alpha_{m}\beta_{m}e^{‐\mu \tau_{m}}v_{1m}\left(0\right)s\left(t_{l}\right)\int_{0}^{T‐t_{l}‐\tau_{m}}e^{‐\frac{\alpha_{m}v_{1m}\left(0\right)e^{‐\delta_{m}\left(u+t_{l}\right)}\left(‐1+e^{\delta_{m}u}\right)}{\delta_{m}}‐\frac{\alpha {\hat{v}}^{*}e^{‐\delta (u+t_{l})}\left(‐1+e^{\delta u}\right)}{\delta }‐\delta_{m}t_{l}‐\mu u}du\right)$$

When $\tau >T‐t_{l}$, mutant invasion fitness can be found using

(3b) $$v_{2m}\left(T\right)=\frac{\alpha_{m}\beta_{m}e^{‐\mu \tau_{m}}v_{1m}\left(0\right){\hat{s}}^{*}}{t_{l}}e^{‐\delta_{m}\left(T‐\tau_{m}\right)}\int_{0}^{T‐\tau_{m}}e^{\left(‐\mu u+\frac{\alpha {\hat{v}}^{*}e^{‐\delta u}}{\delta }+\frac{\alpha_{m}v_{1m}\left(0\right)e^{‐\delta_{m}u}}{\delta_{m}}\right)}\int_{0}^{u}e^{\left(\mu x‐\frac{\alpha {\hat{v}}^{*}e^{‐\delta s}}{\delta }‐\frac{\alpha_{m}v_{1m}\left(0\right)e^{‐\delta_{m}x}}{\delta_{m}}\right)}dxdu$$

To study the evolution of virulence traits in equilibrial environments, we assume that resident and mutant strains are identical at all other traits (e.g., $\alpha =\alpha_{m}$). Note that because there is no trade‐off between *β* and *τ*, the parasite growth rate in the host is the trait under selection. That is, *β* is constant regardless of *τ* such that the time between infection and the release of new parasites is the rate that *β* new parasites are assembled. The uninvadable trait value that maximizes (3) is the optimal virulence level for a given host phenological scenario. That is, the virulence trait $\left(\tau^{*}\right)$ that satisfies

$${\left.\frac{\partial v_{2m}\left(T\right)}{\partial \tau_{m}}\right|}_{\tau_{m}=\tau_{r}}=0$$

$${\left.\frac{\partial^{2}v_{2m}\left(T\right)}{\partial \tau_{m}^{2}}\right|}_{\tau_{m}=\tau_{r}}<0$$

$v_{2m}\left(T\right)$ in equations (3a) and (3b) incorporates the effect of the resident on the population state (number of susceptibles over one season). This means that $v_{2m}\left(T\right)$ is not a measure of *R*_0_, which by definition assumes a nondisease environment. Thus, we can use $v_{2m}\left(T\right)$ as defined in (3a) and (3b) as a maximand in evolutionary dynamics (Lion & Metz, 2018).

In the present study, cycling can occur when host carryover is included in the model for some parameter ranges. When parasite–host dynamics are cycling (3) no longer reliably predicts the outcome of parasite evolution as periods of low host density can drive adaptive mutants to densities less than 1. From a purely mathematical standpoint, the criterion $v_{2m}\left(T\right)\geq 1$ correctly predicts which mutants can invade in cycling populations. However, the invasion criterion does not account for the possibility that a mutant parasite that invades in its first season can drop below 1 in a later season. We thus conduct simulation analysis to verify that the evolutionary stable level of virulence is qualitatively the same as previous results.

The simulation analysis was done by first numerically simulating system (1) with a monomorphic parasite population. A single mutant parasite is introduced at the beginning of the 100th season when the system dynamics have settled on their attractor. The mutant's virulence strategy is drawn from a normal distribution whose mean is the value of *τ* from the resident strain $\left(\tau_{m}=\tau_{r}+N\left(0,0.1\right)\right)$. System (2) is then numerically simulated with the resident and mutant parasite. New mutants arise randomly after 1000 seasons have passed since the last mutant was introduced, at which point system (2) begins following the dynamics of the new parasite strain. This new mutant has a virulence strategy drawn from a normal distribution whose mean is the value of *τ* from whichever parasite strain has the highest density. Note that we decouple mutational input from population size by assuming that mutants arise randomly, regardless of the parasite population size. System (2) follows each new mutant randomly introduced after at least 1000 seasons have passed. Any parasite whose density falls below 1 is considered extinct and is eliminated. Virulence evolves as the population of parasites with the adaptive strategy eventually invade and rise in density. Note that our simulations deviate from the adaptive dynamics literature in that new mutants can be introduced before earlier mutants have replaced the previous resident. Previous studies have shown that this approach is well suited to predicting evolutionary outcomes (Kisdi, 1999; White & Bowers, 2005; White et al., 2006).

## 3 RESULTS

**FIGURE 2:**
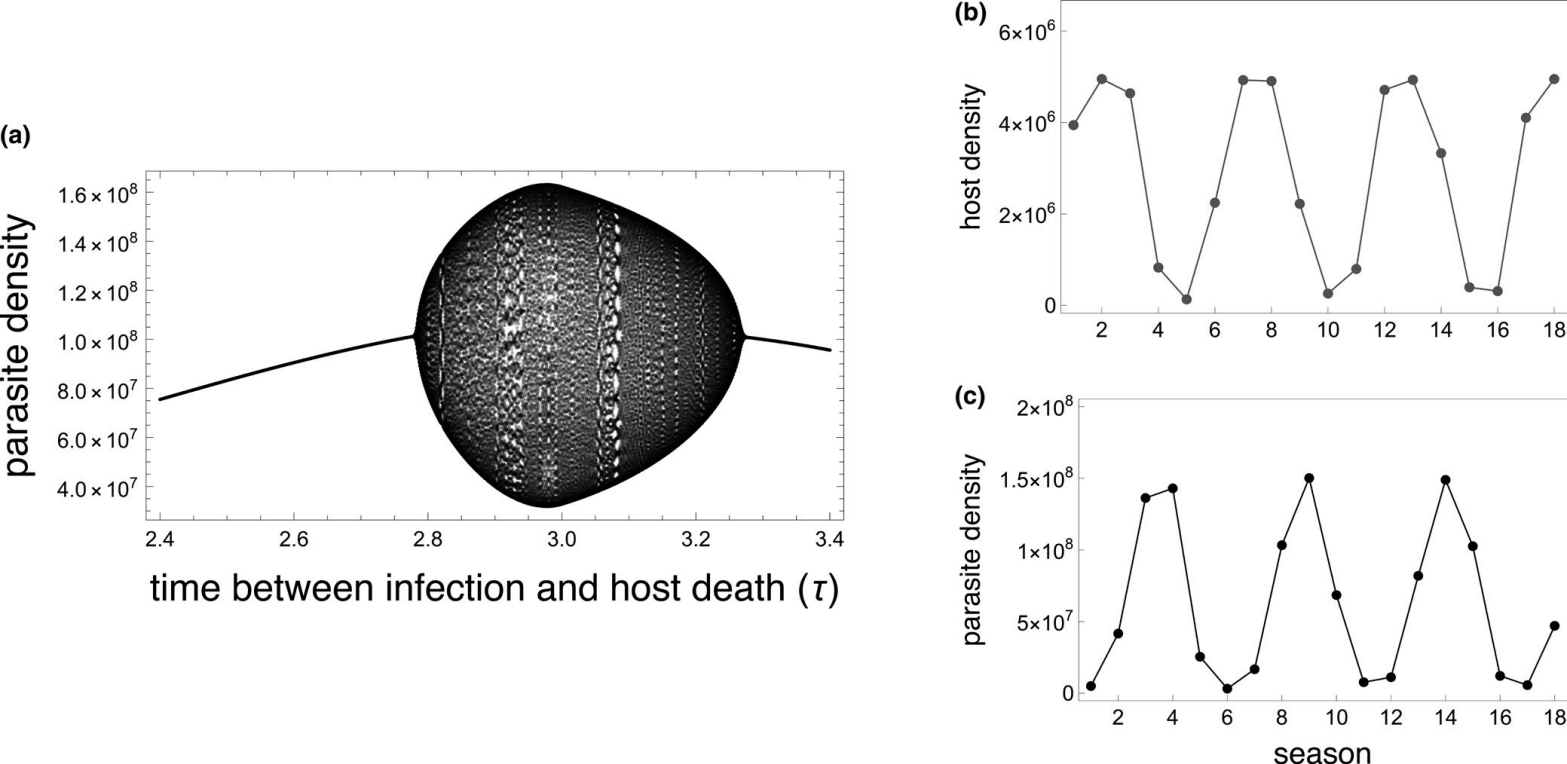
High‐fitness parasites can drive multi‐season epidemic cycles. (a) parasite density increases as the virulence phenotype approaches the time between infection and host death (*τ*) that maximizes parasite fitness (MacDonald et al., 2021). Parasite populations can reach sufficiently high densities in some host phenological patterns, as seen in (a), to destabilize demographic dynamics resulting in a bifurcation that drives quasiperiodic parasite–host dynamics. The bifurcation diagram shows end of season parasite densities for parasites with different virulence phenotypes (*τ*) during seasons 800–900 in a system where the host season is short (T=4) and hosts emerge synchronously ($t_{l}=1$). The most fit parasites (2.75<τ<3.26) achieve densities that can disrupt dynamics and cause cycling. Parasites with virulence phenotypes that are too high (τ<2.75) or too low (τ>3.26) do not cause parasite–host cycles in this host phenological environment. (b‐c) The population dynamics of hosts (b) and parasites (c) in a system experiencing quasiperiodic population cycles ($\tau =2.8,T=4,t_{l}=1$, other parameters found in Table 1) after reaching the quasiperiodic attractor. High parasite densities (ex. season 3–4) infect and sterilize a large proportion of the host population resulting in a dramatic host population decline (ex. seasons 4–5). The limited number of susceptible hosts causes a subsequent decline in parasite populations (ex. seasons 5–7). Host density rebounds once relieved from infection pressure (ex. seasons 6–8) allowing the parasites to exploit the host population again, driving a continuation of quasiperiodic cycling. In both panels: $T=4,t_{l}=1$, all other parameters found in Table 1

Parasites with high fitness in some seasonal environments can drive dynamic parasite–host cycles resembling classical consumer–resource cycles (Figure 2). In the present model, parasites that can achieve sufficiently high densities infect and sterilize a substantial proportion of the univoltine host population resulting in both a decrease in the host population size and an increase in the parasite population size in subsequent seasons. The resulting small host population sizes limit the number of new infections, which leads to a dramatic decrease in parasite population size in the following seasons. Very small parasite populations, in turn, release the host population from parasite‐mediated density control allowing the host population to increase in size. This cycle continues with large host populations supporting rapid parasite population growth, which then drives down the size of the host population. In the current model, one complete cycle requires at least four seasons with parasite population size peaks trailing the host population peaks by two to three seasons.

Parasites adapted to different host phenological patterns reach different densities. As previously demonstrated, parasites adapted to environments with shorter seasons and more synchronous host emergence achieve greater densities than parasites adapted to environments with longer seasons or more variable host emergence timing (MacDonald et al., 2021). In these models, shorter seasons limit the number of infected hosts that die mid‐season due to natural host mortality, resulting in greater parasite population growth rates and greater densities. More synchronous host emergence results in greater numbers of parasites successfully infecting hosts by increasing density‐dependent transmission, thus leading to higher parasite densities. That is, synchronous host emergence results in all infections occurring near simultaneously such that adapted parasites will kill all infected hosts near the end of the season in order to minimize decay of parasite progeny in the environment. By contrast, parasites in environments with greater host emergence variation have virulence levels that cause hosts infected early in the season to release progeny too early—where parasites decay in the environment—and to not kill hosts infected later in the season where hosts die naturally without producing parasite progeny. Thus, short seasons and synchronous host emergence both increase parasite density by reducing parasite mortality either through infected‐host mortality or through environmental decay.

**FIGURE 3:**
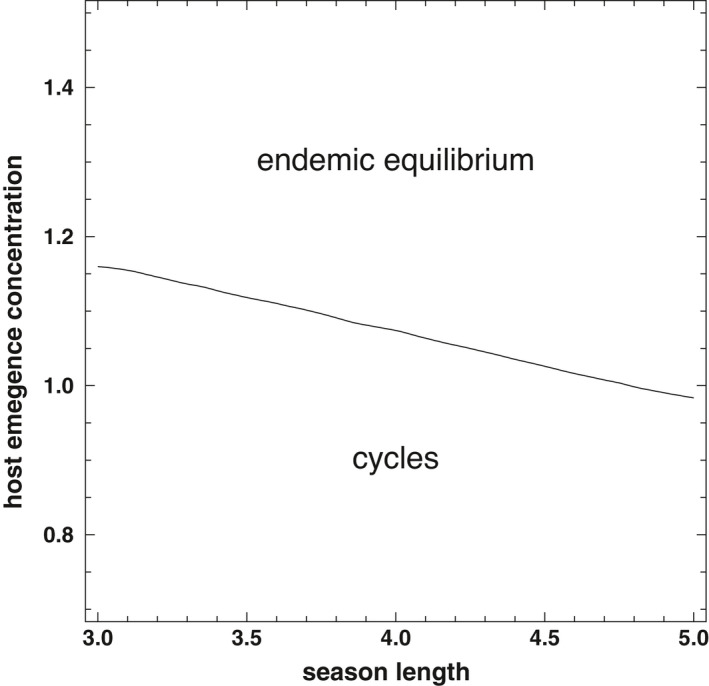
Parasite–host cycles occur in some, but not all, host phenological patterns. Boundary plot shows host phenological patterns where dynamics are stable (“endemic equilibrium”) or cycling for parasites possess the optimal virulence trait for their phenological environment. Parasites are more likely to achieve the densities necessary to drive cycles when host emergence periods are short (small values of *T*) and host emergence is synchronous (small values of *t_l_*). All other parameters found in Table 1

Host phenological patterns influence parasite densities and thus if cycling occurs. For example, parasites in environments with shorter host activity seasons can reach sufficiently high densities to provoke host–parasite population cycles (Figure 3). By contrast, long seasons prevent population cycles by limiting parasite densities below levels that destabilize host–parasite dynamics (Figure 3). Parasites in phenological environments with limited variation in the time when each host first emerges within a season are also more likely to achieve population cycle‐inducing densities than environments with more variable host emergence timing (Figure 3). The loss of potential parasite progeny through environmental decay or infected‐host mortality of infected hosts limits parasite density to levels below those that can destabilize host–parasite dynamics and cause demographic cycles.

**FIGURE 4:**
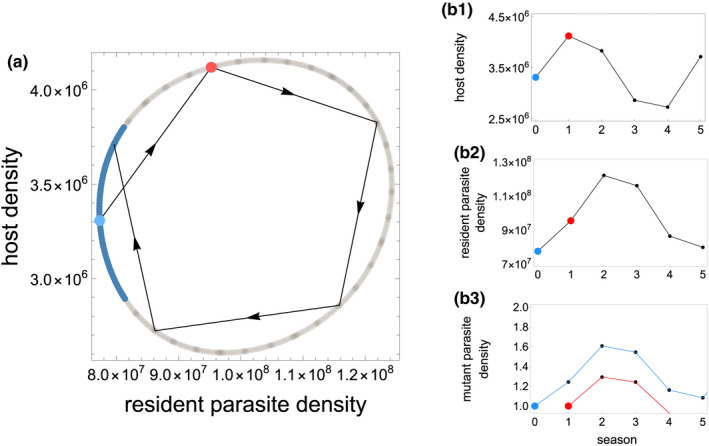
Mutant parasites with more adaptive virulence phenotypes often fail to invade when resident parasite and host dynamics are cycling. The phase plane (a) shows the discrete time limit cycle for host ($\hat{s}$) and resident parasite ($\hat{v}$) densities ($T=4;t_{l}=1;\tau =2.8$ in this example). The blue section denotes the phase of the parasite–host cycle when rare adaptive parasites can invade; the same mutant fails to invade when introduced at all other time points despite having the same selective advantage. The line (a) depicts the same iteration (six seasons) of the quasiperiodic dynamics of this system as illustrated in (b1) and (b2). An advantageous mutant fails to invade (red line, b3) if introduced in seasons when host density will decrease (red point, b1) and resident parasite density is moderate or high (red point, b2). The same advantageous mutant can invade (blue line, b3) and eventually replace the resident parasite if it is introduced when host density will increase (blue point, b1) and resident parasite density is low (blue point, b2). $\tau_{m}=2.81$, all other parameters found in Table 1

The parasite densities that can be attained in each host phenological scenario determines whether the system reaches stable equilibrial inter‐annual dynamics or quasiperiodic parasite–host population cycles (Figure 3). In the majority of scenarios in which cycling occurs, the discrete dynamics form a closed invariant curve in the phase plane in which the phase is incommensurate, and thus, the asymptotic trajectory fills the invariant curve by never repeating itself (Figure 4a). That is, the population sizes of both the host and parasite do not repeat across seasons, resulting in quasiperiodic cycles that are likely generated by a Neimark–Sacker bifurcation (Strogatz, 2018; see Appendix S1).

Additional environmental factors that promote high parasite density, such as low environmental decay rates, can increase the parameter region where cycling occurs (Table 2). Higher parasite densities result in more synchronous infections early in the season leading to greater parasite densities and a greater likelihood that parasites destabilize host dynamics. Conversely, conditions that limit parasite density, such as greater natural host mortality rates, decrease the parameter range where cycling occurs. Higher host mortality rates increase the death rate of infected hosts and thus decrease the number of infections that successfully release parasite progeny. When fewer infections release new parasites, the parasite population is less likely to reach densities that can generate cycles.

**TABLE 2:** The impact of each variable on whether or not demographic cycles occur

Increases in variable value	Impact on possibility of demographic cycling
Season length (*T*)	↓ cycling
Emergence period length (*t_l_*)	↓ cycling
Host mortality (*d*)	↓ cycling
Decay rate (*δ*)	↓ cycling
Transmission rate (*α*)	↑ cycling
Parasites released at host death (*β*)	↑ cycling
Host fecundity (*σ*)	↑ cycling

**FIGURE 5:**
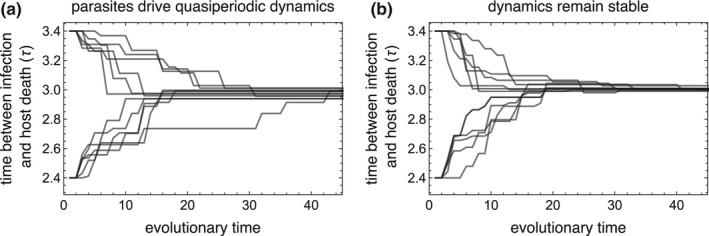
Adaptive evolution proceeds more slowly during parasite–host demographic cycles than in stable equilibrial systems. Parasite evolution toward an intermediate optimal virulence strategy occurs more slowly when population demography is cycling (a) than in an equilibrial dynamic system (b). (a) Increases in parasite density as parasites evolve drive demographic cycling for 2.75<τ<3.26. Population cycling delays adaptive evolution as rare mutants fail to invade the system when introduced at many phases of the dynamic cycle despite their selective advantage. By contrast, rare advantageous mutants always invade systems with stable dynamics (b). Plots show twelve independent simulations for each set of parameters—six runs starting at a virulence level lower than optimum and six runs starting at a virulence level higher than the optimum—where an adaptive mutant is introduced into the population no more than once every 1000 seasons. Evolutionary time represents the cumulative number of adaptive mutants sequentially introduced into each population. The average evolutionary time needed to reach the optimal virulence strategy is higher in the cycling system ((a) 21 mutants, range: 6–42 mutants) than in the stable system (b. 14 mutants, range: 6–27 mutants). Population cycling could not occur in (b) as the host cohort size remained constant across seasons ($\hat{s}=10^{8}$); host cohort size in (a) $\left(\hat{s}\left(n\right)\right)$ was determined by the number of hosts that reproduced in season n‐1. $T=4,t_{l}=1,$ all other parameters found in Table 1

Parasite–host population cycles impede the rate at which parasite traits adapt to host phenological environments (Figures 4 and 5). Rare advantageous mutations readily invade systems in which the populations are not cycling. However, the phase of a population cycle at which a rare advantageous mutant is introduced into a system determines whether it will displace the resident parasite. Rare advantageous mutants invade cycling systems only in seasons when the resident parasite population is at a low density, and the host population size is increasing or is at a high density. By contrast, novel advantageous mutants often fail to establish when resident parasites are at a high density because high parasite densities drive a rapid host density decrease in the following seasons, resulting in limited parasite resources and a parasite population bottleneck. The parasite population bottleneck often drives extinctions of the rare advantageous mutant parasite strain. This eco‐evolutionary feedback results in the extinction of many advantageous mutants and a reduced rate of evolution toward the virulence strategy that optimizes parasite fitness.

## 4 DISCUSSION

Host phenological patterns govern parasite densities directly through the timing and frequency of ecological interactions, which can lead to an over‐exploitation of hosts and subsequent parasite–host population cycles. Parasites can achieve sufficiently high densities in only some host phenological environments to destabilize the host–parasite dynamics that instigate quasiperiodic population cycles. The population cycles result from the classic consumer–resource ecological feedback where the parasite consumer overexploits their host resource such that the host population cannot fully recover in the following year. The resulting host population size is insufficient to support the now excessively large parasite population, which results in a dramatic decline in the number of parasites in the following year. The host population can then rebound due to the limited demographic impact of parasitism, thus allowing parasites to again over‐exploit their hosts and restart the population cycle. An evolutionary feedback can also result from this consumer–resource ecological feedback. Parasite adaptation toward optimal trait values proceeds more slowly when host–parasite dynamics are cycling. That is, many mutant parasites with adaptive phenotypes that arise in a cycling system will not increase in frequency and ultimately be lost from the population.

The observed parasite–host population cycles emerge from a delayed density‐dependent mechanism characteristic of consumer–resource feedbacks (Turchin, 2013). In this system, the discrete host activity period introduces a delayed carryover effect in which the number of infected hosts in one season governs the host population size in the next. Although consumer–resource interactions can drive cycling in continuous time models, cycles are less likely to occur without an externally imposed delay (Keeling & Rohani, 2011). The results of this study differ from those of prior studies describing consumer–resource feedbacks as this delayed density‐dependent mechanism causes population cycles only in phenological environments that support high parasite densities. Phenological patterns where hosts have shorter seasons and more synchronous emergence limit parasite deaths caused by environmental decay and infected‐host deaths, thus resulting in large parasite populations that can destabilize parasite–host dynamics and cause population cycles. By contrast, longer seasons and greater variation in emergence times among hosts support lower parasite densities, which do not cause population cycles.

The stable parasite–host dynamics observed in some host phenological patterns differs from seminal theoretical studies demonstrating chaotic dynamics at all population growth rates of lethal parasites (May, 1985). Our results suggest that host phenology can stabilize host–parasite dynamics and provide one potential explanation for why chaotic dynamics are often not observed in natural obligate‐killer parasite systems. Other model parameters such as natural host mortality rate and parasite decay rate also modulate parasite population sizes and thus also alter which phenological scenarios can lead to periodic population cycles. Furthermore, several factors that have been shown to impact the probability of dynamic population cycles not explored in this model could also modulate the phenological scenarios in which cycling could be expected (Hilker et al., 2020; Koella & Doebeli, 1999). For example, higher infected‐host fecundity would likely stabilize the dynamics for a greater range of phenological patterns.

Population cycles resulting from a consumer–resource ecological feedback precipitates an eco‐evolutionary feedback that affects the rate of adaptive evolution. In this model, parasites with advantageous mutations always invade non‐cycling systems. That is, advantageous mutants displace residents both in systems where the parasite is not sufficiently adapted in a host phenological environment that could support high parasite densities as well as in systems where the host phenological pattern cannot support densities sufficient to cause population cycling even for optimally adapted parasites. By contrast, only a fraction of parasites with adaptive mutations introduced into cycling systems can invade, effectively reducing the rate of adaptive evolution. These results suggests that adaptation in cycling seasonal disease systems is likely to proceed more slowly. Parasites with adaptive mutations that do not invade fail to increase sufficiently to prevent their extinction due to the parasite population bottleneck that results from rapid decreases in host density. This result echos results from invasion ecology demonstrating that the timing of introduction predicts invasion success by creating or destroying niche space for invader prey species in a cycling predator–prey model (Yamamichi et al., 2014). An assumption of the current model is that mutants are introduced at the beginning of random seasons, regardless of parasite population size. However, mutants are less likely to arise when parasite population size is small (Crow & Kimura, 1970) suggesting that the true impact of population cycling on the evolutionary rate is likely greater than estimated here. That is, the proportion of advantageous mutants lost in cycling populations in nature is likely greater than found here as mutants are more likely to arise at points in the cycle when parasite populations are large and on the precipice of crashing.

Our results extend previous theory on the interaction between cycling and the rate of adaptive evolution. Many previously published investigations focus on the impact of temporal fluctuations on long‐term evolutionary outcomes (e.g., Donnelly et al., 2013; Ferriere & Gatto, 1995; Ferris & Best, 2018; Grunert et al., 2021; Metz et al., 1992). The results presented here suggest that temporal cycling can slow the rate of adaptive evolution by constraining when adaptive mutants can successfully invade, even if the long‐term evolutionary outcome remains constant. That is, while prior work revealed the most advantageous long‐term evolutionary strategies (Ferriere & Gatto, 1995; Metz et al., 1992), our approach identified the demographic conditions leading to the extinction of advantageous mutants. In addition, our results support previously published conclusions showing that evolutionary adaptions can not only drive demographic cycles (Ferriere & Gatto, 1993; Metz et al., 1995) but also extends this work through our result that cycling slows the rate of adaptive evolution.

These results suggest that spatial variation in host phenology could drive differences in demographic dynamics observed across geographic space. For example, parasite–host systems in more extreme latitudes and at higher altitudes are more likely to cycle than conspecifics in less extreme environments (Baltensweiler & Fischlin, 1988; Klemola et al., 2002; Schott et al., 2010). The activity periods in the more extreme environments tend to be shorter, and hosts may emerge more synchronously (Inouye & Wielgolaski, 2013; Wielgolaski & Inouye, 2013) in line with the predictions from the current model. These predictions could be tested empirically by studying the population dynamics of disease systems with forest *Lepidoptera* hosts in different geographic locations. Key parasite traits such as the parasite latency period could also be measured to determine how parasite adaptation to different phenological patterns affected the differing demographic dynamics. Empirical data across locations could examine how phenology impacts species interactions and how that could cause differences in population densities, selection, and dynamical trajectories.

Several features of the current model can be altered to investigate more complex impacts of host phenology on parasite–host dynamics and eco‐evolutionary feedbacks. For example, permitting host evolution in either parasite resistance or phenological patterns could drive additional eco‐evolutionary feedbacks through changes in the strength of selection imposed on hosts by parasite infections (Best, 2018; Ferris et al., 2020). Future theoretical and empirical investigations into the impact of parasite–host cycles on the evolution of host resistance alleles, as seen in Gypsy moth populations (Elderd et al., 2008), could determine whether parasite–host co‐evolution would stabilize population dynamics for a greater range of host phenological patterns. Similarly, the strength and possibly direction of selection on hosts will fluctuate as the system cycles, potentially favoring alternative host phenological patterns that in turn select for parasite traits with lower impacts on host fitness. Another interesting extension is the role genetic drift could play for parasite adaptation in stable versus cycling dynamics (Kennedy & Dwyer, 2018). The impact of drift on parasite evolution in cycling populations is highly complex and difficult to predict *a priori*. We will extend the current model to incorporate neutral evolution in future studies.

Relaxing some of the assumptions in this model is unlikely to qualitatively alter the major conclusions. For example, relaxing the monocylic parasite life cycle assumption will likely not change the result that cycles occur more readily in environments with short seasons and synchronous host emergence. Polycyclic parasites may even drive cycles for a larger range of phenological patterns as multiple infection cycles within a season can exacerbate decreases in host densities. Similarly, relaxing the obligate‐killer assumption will likely decrease but not eliminate the range of phenological patterns that drive cycles by decreasing the impact on host fitness. Although the model as presented applies to only a narrow range of parasites in nature, many more parasite–host systems conform to models that include these extensions such as soil‐borne plant pathogens, demicyclic rusts, postharvest diseases, and many diseases infecting univoltine insects (Crowell, 1934; Gaulin et al., 2007; Holuša & Lukášová, 2017; Zehr, 1982).

Environmental conditions such as phenology impact the frequency of interspecies interactions and thus the ecological importance of the interaction on population demography. Here, we show that short host seasons and synchronous host emergence allow parasites to reach densities sufficient to destabilize population dynamics and cause demographic cycling. The rate of adaptive parasite evolution in a cycling population is substantially slower than in an equilibrial population as beneficial mutations are more likely to go extinct when host population sizes are small or parasite population sizes are large. These results demonstrate that externally imposed environmental conditions such as host phenology can be important determinants of population cycling. It is important to consider ecological dynamics when predicting evolution by natural selection.

## CONFLICT OF INTEREST

The authors declare no conflicts of interest.

## AUTHOR CONTRIBUTION

**Hannelore MacDonald involved in** conceptualization (lead), formal analysis (lead), investigation (lead), visualization (lead), writing—original draft (lead), and writing—review and editing (equal). **Dustin Brisson involved in** funding acquisition (lead), investigation (supporting), writing—original draft (supporting), and writing—review and editing (equal).

## Supporting information

Appendix S1‐S2

## Data Availability

Code is available on the Github repository: https://github.com/hanneloremac/Host‐phenology‐regulates‐parasite‐host‐demographic‐cycles‐and‐eco‐evolutionary‐feedbacks.

## References

[ece38658-bib-0001] Abbott, K. C. , & Dwyer, G. (2007). Food limitation and insect outbreaks: Complex dynamics in plantherbivore models. Journal of Animal Ecology, 1004–1014. 10.1111/j.1365-2656.2007.01263.x 17714279

[ece38658-bib-0002] Anderson, R. M. , & May, R. M. (1981). The population dynamics of microparasites and their invertebrate hosts. Philosophical Transactions of the Royal Society of London. B, Biological Sciences, 291(1054), 451–524. 10.1098/rstb.2014.0307 PMC4360116 25750231

[ece38658-bib-0003] Baltensweiler, W. , & Fischlin, A. (1988). The larch budmoth in the alps. In A. A. Berryman (Ed.), Dynamics of forest insect populations (pp. 331–351). Springer.

[ece38658-bib-0004] Barber, I. , Berkhout, B. W. , & Ismail, Z. (2016). Thermal change and the dynamics of multi‐host parasite life cycles in aquatic ecosystems. Integrative and Comparative Biology, 56(4), 561–572. 10.1093/icb/icw025 27252219 PMC5035383

[ece38658-bib-0005] Best, A. (2018). Host–pathogen coevolution in the presence of predators: Fluctuating selection and ecological feedbacks. Proceedings of the Royal Society B, 285(1885), 20180928. 30135155 10.1098/rspb.2018.0928 PMC6125909

[ece38658-bib-0006] Bewick, S. , Cantrell, R. S. , Cosner, C. , & Fagan, W. F. (2016). How resource phenology affects consumer population dynamics. The American Naturalist, 187(2), 151–166. 10.1086/684432 26807744

[ece38658-bib-0007] Burkett‐Cadena, N. D. , McClure, C. J. , Ligon, R. A. , Graham, S. P. , Guyer, C. , Hill, G. E. , Ditchkoff, S. S. , Eubanks, M. D. , Hassan, H. K. , & Unnasch, T. R. (2011). Host reproductive phenology drives seasonal patterns of host use in mosquitoes. PLoS One, 6(3), e17681. 10.1371/journal.pone.0017681 21408172 PMC3049777

[ece38658-bib-0008] Campbell, R. W. (1975). The gypsy moth and its natural enemies. Number 381. US Department of Agriculture, Forest Service.

[ece38658-bib-0009] Caraco, T. , & Wang, I.‐N. (2008). Free‐living pathogens: Life‐history constraints and strain competition. Journal of Theoretical Biology, 250(3), 569–579. 18062992 10.1016/j.jtbi.2007.10.029 PMC2262931

[ece38658-bib-0010] Crow, J. F. , & Kimura, M. (1970). An introduction to population genetics theory. Population, 26(5), 977.

[ece38658-bib-0011] Crowell, I. H. (1934). The hosts, life history and control of the cedar‐apple rust fungus gymnosporangium juniperi‐virginianae schw. Journal of the Arnold Arboretum, 15(3), 163–232. 10.5962/p.185310

[ece38658-bib-0012] Delucchi, V. (1982). Parasitoids and hyperparasitoids of zeiraphera diniana [lep., tortricidae] and their pole in population control in outbreak areas. Entomophaga, 27(1), 77–92. 10.1007/BF02371940

[ece38658-bib-0013] Donnelly, R. , Best, A. , White, A. , & Boots, M. (2013). Seasonality selects for more acutely virulent parasites when virulence is density dependent. Proceedings of the Royal Society B: Biological Sciences, 280(1751), 20122464. 10.1098/rspb.2012.2464 PMC3574413 23193133

[ece38658-bib-0014] Dwyer, G. (1994). Density dependence and spatial structure in the dynamics of insect pathogens. The American Naturalist, 143(4), 533–562. 10.1086/285619

[ece38658-bib-0015] Elderd, B. D. , Dushoff, J. , & Dwyer, G. (2008). Host‐pathogen interactions, insect outbreaks, and natural selection for disease resistance. The American Naturalist, 172(6), 829–842. 10.1086/592403 18976065

[ece38658-bib-0016] Ferriere, R. , & Gatto, M. (1993). Chaotic population dynamics can result from natural selection. Proceedings of the Royal Society of London. Series B: Biological Sciences, 251(1330), 33–38. 8094563 10.1098/rspb.1993.0005

[ece38658-bib-0017] Ferriere, R. , & Gatto, M. (1995). Lyapunov exponents and the mathematics of invasion in oscillatory or chaotic populations. Theoretical Population Biology, 48(2), 126–171. 10.1006/tpbi.1995.1024

[ece38658-bib-0018] Ferris, C. , & Best, A. (2018). The evolution of host defence to parasitism in fluctuating environments. Journal of Theoretical Biology, 440, 58–65. 10.1016/j.jtbi.2017.12.006 29221891

[ece38658-bib-0019] Ferris, C. , Wright, R. , Brockhurst, M. A. , & Best, A. (2020). The evolution of host resistance and parasite infectivity is highest in seasonal resource environments that oscillate at intermediate amplitudes. Proceedings of the Royal Society B: Biological Sciences, 287(1927), 20200787. 10.1098/rspb.2020.0787 PMC7287369 32453992

[ece38658-bib-0020] Fine, P. E. , & Clarkson, J. A. (1982). Measles in England and wales—I: An analysis of factors underlying seasonal patterns. International Journal of Epidemiology, 11(1), 5–14. 7085179 10.1093/ije/11.1.5

[ece38658-bib-0021] Finkenstädt, B. F. , & Grenfell, B. T. (2000). Time series modelling of childhood diseases: A dynamical systems approach. Journal of the Royal Statistical Society: Series C, 49(2), 187–205.

[ece38658-bib-0022] Gaulin, E. , Jacquet, C. , Bottin, A. , & Dumas, B. (2007). Root rot disease of legumes caused by *Aphanomyces euteiches* . Molecular Plant Pathology, 8(5), 539–548. 10.1111/j.1364-3703.2007.00413.x 20507520

[ece38658-bib-0023] Geritz, S. , Kisdi, É. , Meszéna, G. , & Metz, J. (1998). Evolutionarily singular strategies and the adaptive growth and branching of the evolutionary tree. Evolutionary Ecology, 12(1), 35–57. 10.1023/A:1006554906681

[ece38658-bib-0024] Govaert, L. , Fronhofer, E. A. , Lion, S. , Eizaguirre, C. , Bonte, D. , Egas, M. , Hendry, A. P. , De Brito Martins, A. , Melián, C. J. , Raeymaekers, J. A. M. , Ratikainen, I. I. , Saether, B.‐E. , Schweitzer, J. A. , & Matthews, B. (2019). Eco‐evolutionary feedbacks—Theoretical models and perspectives. Functional Ecology, 33(1), 13–30. 10.1111/1365-2435.13241

[ece38658-bib-0025] Greenman, J. , Kamo, M. , & Boots, M. (2004). External forcing of ecological and epidemiological systems: A resonance approach. Physica D: Nonlinear Phenomena, 190(1–2), 136–151.

[ece38658-bib-0026] Grunert, K. , Holden, H. , Jakobsen, E. R. , & Stenseth, N. C. (2021). Evolutionarily stable strategies in stable and periodically fluctuating populations: The Rosenzweig–Macarthur predator–prey model. Proceedings of the National Academy of Sciences, 118(4), 2021. 10.1073/pnas.2017463118 PMC7848735 33479183

[ece38658-bib-0027] Hilker, F. , Sun, T. , Allen, L. , & Hamelin, F. (2020). Separate seasons of infection and reproduction can lead to multi‐year population cycles. Journal of Theoretical Biology, 489, 110158. 10.1016/j.jtbi.2020.110158 31926973

[ece38658-bib-0028] Hite, J. L. , & Cressler, C. E. (2018). Resource‐driven changes to host population stability alter the evolution of virulence and transmission. Philosophical Transactions of the Royal Society B: Biological Sciences, 373(1745), 20170087. 10.1098/rstb.2017.0087 PMC5882993 29531142

[ece38658-bib-0029] Holuša, J. , & Lukášová, K. (2017). Pathogen’s level and parasitism rate in ips typographus at high population densities: Importance of time. Journal of Applied Entomology, 141(9), 768–779.

[ece38658-bib-0030] Inouye, D. W. , & Wielgolaski, F. E. (2013). Phenology at high altitudes. In M. D. Schwartz (Ed.), Phenology: An integrative environmental science (pp. 249–272). Springer.

[ece38658-bib-0031] Kamo, M. , & Sasaki, A. (2002). The effect of cross‐immunity and seasonal forcing in a multi‐strain epidemic model. Physica D: Nonlinear Phenomena, 165(3–4), 228–241. 10.1016/S0167-2789(02)00389-5

[ece38658-bib-0032] Keeling, M. J. , & Rohani, P. (2011). Modeling infectious diseases in humans and animals. Princeton University Press.

[ece38658-bib-0033] Kenis, M. , & Hilszczanski, J. (2007). Natural enemies of cerambycidae and buprestidae infesting living trees. In F. Lieutier , K. R. Day , A. Battisti , J. C. Grégoire , & H. F. Evans (Eds.), Bark and wood boring insects in living trees in Europe, a synthesis (pp. 475–498). Springer.

[ece38658-bib-0034] Kennedy, D. A. , & Dwyer, G. (2018). Effects of multiple sources of genetic drift on pathogen variation within hosts. PLoS Biology, 16(3), e2004444. 10.1371/journal.pbio.2004444 29590105 PMC5891033

[ece38658-bib-0035] Kisdi, E. (1999). Evolutionary branching under asymmetric competition. Journal of Theoretical Biology, 197(2), 149–162. 10.1006/jtbi.1998.0864 10074390

[ece38658-bib-0036] Klemola, T. , Tanhuanpää, M. , Korpimäki, E. , & Ruohomäki, K. (2002). Specialist and generalist natural enemies as an explanation for geographical gradients in population cycles of northern herbivores. Oikos, 99(1), 83–94. 10.1034/j.1600-0706.2002.990109.x

[ece38658-bib-0037] Koella, J. C. , & Doebeli, M. (1999). Population dynamics and the evolution of virulence in epidemiological models with discrete host generations. Journal of Theoretical Biology, 198(3), 461–475. 10.1006/jtbi.1999.0925 10366497

[ece38658-bib-0038] Krebs, C. J. (2013). Population fluctuations in rodents. University of Chicago Press.

[ece38658-bib-0039] Lion, S. , & Metz, J. A. (2018). Beyond r0 maximisation: On pathogen evolution and environmental dimensions. Trends in Ecology & Evolution, 33(6), 458–473. 29665966 10.1016/j.tree.2018.02.004

[ece38658-bib-0040] MacDonald, H. , Akçay, E. , & Brisson, D. (2021). Host phenology can drive the evolution of intermediate virulence strategies in some parasites. bioRxiv. 10.1101/2021.03.13.435259. PMC9540771 35488459

[ece38658-bib-0041] May, R. M. (1985). Regulation of populations with nonoverlapping generations by microparasites: A purely chaotic system. The American Naturalist, 125(4), 573–584.

[ece38658-bib-0042] Metz, J. A. , Geritz, S. A. , Meszéna, G. , Jacobs, F. J. , & Van Heerwaarden, J. S. (1995). Adaptive dynamics: A geometrical study of the consequences of nearly faithful reproduction. International Institute for Applied Systems Analysis.

[ece38658-bib-0043] Metz, J. A. , Nisbet, R. M. , & Geritz, S. A. (1992). How should we define ‘fitness’ for general ecological scenarios? Trends in Ecology & Evolution, 7(6), 198–202. 10.1016/0169-5347(92)90073-K 21236007

[ece38658-bib-0044] Miller‐Rushing, A. J. , Høye, T. T. , Inouye, D. W. , & Post, E. (2010). The effects of phenological mismatches on demography. Philosophical Transactions of the Royal Society B: Biological Sciences, 365(1555), 3177–3186. 10.1098/rstb.2010.0148 PMC2981949 20819811

[ece38658-bib-0045] Myers, J. H. (2018). Population cycles: Generalities, exceptions and remaining mysteries. Proceedings of the Royal Society B: Biological Sciences, 285(1875), 20172841. 10.1098/rspb.2017.2841 PMC5897639 29563267

[ece38658-bib-0046] Myers, J. H. , & Cory, J. S. (2013). Population cycles in forest lepidoptera revisited. Annual Review of Ecology, Evolution, and Systematics, 44, 565–592. 10.1146/annurev-ecolsys-110512-135858

[ece38658-bib-0047] Paull, S. H. , & Johnson, P. T. (2014). Experimental warming drives a seasonal shift in the timing of host‐parasite dynamics with consequences for disease risk. Ecology Letters, 17(4), 445–453. 10.1111/ele.12244 24401007

[ece38658-bib-0048] Schott, T. , Hagen, S. B. , Ims, R. A. , & Yoccoz, N. G. (2010). Are population outbreaks in sub‐arctic geometrids terminated by larval parasitoids? Journal of Animal Ecology, 79(3), 701–708. 10.1111/j.1365-2656.2010.01673.x 20233259

[ece38658-bib-0049] Strogatz, S. H. (2018). Nonlinear dynamics and chaos with student solutions manual: With applications to physics, biology, chemistry, and engineering. CRC Press.

[ece38658-bib-0050] Taylor, R. A. , White, A. , & Sherratt, J. A. (2013). How do variations in seasonality affect population cycles? Proceedings of the Royal Society B: Biological Sciences, 280(1754), 20122714. 10.1098/rspb.2012.2714 PMC3574328 23325773

[ece38658-bib-0051] Turchin, P. (2013). Complex population dynamics. Princeton University Press.

[ece38658-bib-0052] van Asch, M. , & Visser, M. E. (2007). Phenology of forest caterpillars and their host trees: The importance of synchrony. Annual Review of Entomology, 52, 37–55. 10.1146/annurev.ento.52.110405.091418 16842033

[ece38658-bib-0053] Wang, I.‐N. (2006). Lysis timing and bacteriophage fitness. Genetics, 172(1), 17–26. 10.1534/genetics.105.045922 16219778 PMC1456144

[ece38658-bib-0054] White, A. , & Bowers, R. G. (2005). Adaptive dynamics of lotka–volterra systems with trade‐offs: The role of interspecific parameter dependence in branching. Mathematical Biosciences, 193(1), 101–117. 15681278 10.1016/j.mbs.2004.10.006

[ece38658-bib-0055] White, A. , Greenman, J. , Benton, T. , & Boots, M. (2006). Evolutionary behaviour in ecological systems with trade‐offs and non‐equilibrium population dynamics. Evolutionary Ecology Research, 8(3), 387–398.

[ece38658-bib-0056] White, N. J. (2011). Determinants of relapse periodicity in plasmodium vivax malaria. Malaria Journal, 10(1), 1–36. 10.1186/1475-2875-10-297 21989376 PMC3228849

[ece38658-bib-0057] Wielgolaski, F. E. , & Inouye, D. W. (2013). Phenology at high latitudes. In M. D. Schwartz (Ed.), Phenology: an integrative environmental science (pp. 225–247). Springer.

[ece38658-bib-0058] Yamamichi, M. , Yoshida, T. , & Sasaki, A. (2014). Timing and propagule size of invasion determine its success by a time‐varying threshold of demographic regime shift. Ecology, 95(8), 2303–2315. 10.1890/13-1527.1 25230480

[ece38658-bib-0059] Yang, L. H. , & Rudolf, V. (2010). Phenology, ontogeny and the effects of climate change on the timing of species interactions. Ecology Letters, 13(1), 1–10. 10.1111/j.1461-0248.2009.01402.x 19930396

[ece38658-bib-0060] Zehr, E. I. (1982). Control of brown rot in peach orchards. Plant Disease, 66(12), 1101–1105. 10.1094/PD-66-1101

