# Supplementary material for: Host phenology regulates parasite–host demographic cycles and eco‐evolutionary feedbacks
Source: Ecol Evol. 2022 Mar 16;12(3):e8658. doi: 10.1002/ece3.8658 (PMC8928868; doi:10.1002/ece3.8658)
Supplement: Supplementary file 1 — Appendix S1‐S2 [file ECE3-12-e8658-s001.pdf]

Appendix A

In Appendix A we find analytical solutions for equations 1a-c from the main text to study parasite fitness given different host phenological patterns.

$$\frac{ds}{dt} = \hat{s}(n)g(t,t_l) - (t) - \alpha s(t)v_1(t), \tag{A.1a}$$

$$\frac{dv_1}{dt} = -\delta v_1(t), \tag{A.1b}$$

$$\frac{dv_2}{dt} = \alpha\beta e^{-\mu\tau}s(t-\tau)v_1(t-\tau) - \delta v_2(t). \tag{A.1c}$$

with initial conditions: $s(0) = 0, v_1(0^+) = v_2(0^-) = \hat{v}(n), v_2(\tau) = 0$.

(A.1a-c) is solved analytically by describing host emergence using a uniform distribution

$$g(t,t_l) = \begin{cases} \frac{1}{t_l} & 0 \leq t \leq t_l \\ 0 & t_l < t \end{cases}$$

We then use $v_1(t)$ to find the time-dependent solution for $s(t)$. We can then plug the time-dependent solution for $s(t)$ to find the time-dependent solution for $v_2(t)$. Only parasites that infect hosts from $0 < t < T - \tau$ have enough time to kill hosts and release progeny before the end of the season. For $\tau < T - t_l$, parasites that infect hosts during host emergence ($0 < t \leq t_l$) have time to kill hosts and release progeny before the end of the season as well as some parasites who infect hosts after host emergence has ended ($t > t_l$). For $\tau > T - t_l$, only some parasites that infect hosts during host emergence ($0 < t \leq t_l$) have time to kill hosts and release progeny before the end of the season. Thus, two separate solutions are required depending on whether τ is greater or less than $T - t_l$.

We first consider the case where $\tau < T - t_l$:

$$s(t) = \begin{cases} \frac{\hat{s}(n)}{t_l} e^{(-\mu t + \frac{\alpha \hat{v}(n) e^{-\delta t}}{\delta})} \int_0^t e^{(\mu u - \frac{\alpha \hat{v}(n) e^{-\delta u}}{\delta})} du & 0 < t < t_l \\ s(t_l) e^{(-\mu(t - tl) - \frac{\alpha \hat{v}(n) e^{-\delta(t+t_l)}(-1+e^{\delta t})}{\delta})} & t_l \leq t < T \end{cases}$$

$$v_2(t) = \begin{cases} \frac{\alpha \beta e^{-\mu\tau} \hat{v}(n) \hat{s}(n)}{t_l} e^{-\delta(t-\tau)} \int_0^{t-\tau} e^{(-\mu u + \frac{\alpha \hat{v}(n) e^{-\delta u}}{\delta})} \int_0^u e^{(\mu x - \frac{\alpha \hat{v}(n) e^{-\delta x}}{\delta})} dx du & \tau < t < t_l \\ e^{-\delta(t - t_l - \tau)} (v_2(t_l) + \alpha \beta e^{-\mu\tau} \hat{v}(n) s(t_l) \int_0^{t - t_l - \tau} e^{-\frac{\alpha \hat{v}(n) e^{-\delta(u+t_l)}(-1+e^{\delta u})}{\delta} - \delta t_l - \mu u} du) & t_l \leq t < T \end{cases}$$

where $s(t_l)$ and $v_2(t_l)$ are the densities of s and v_2 when the emergence period of s ends.

For $\tau > T - t_l$, only some of the parasites that infect hosts from $0 < t < t_l$ have enough time to kill hosts and release progeny before the end of the season. $v_2(t)$ are thus only produced from infections that occurred from $0 < t < t_l$. The solution for $v_2(t)$ in this case is

$$v_2(t) = \frac{\alpha \beta e^{-\mu\tau} \hat{v}(n) \hat{s}(n)}{t_l} e^{-\delta(t-\tau)} \int_0^{t-\tau} e^{(-\mu u + \frac{\alpha \hat{v}(n) e^{-\delta u}}{\delta})} \int_0^u e^{(\mu x - \frac{\alpha \hat{v}(n) e^{-\delta x}}{\delta})} dx du \qquad \tau < t < T$$

Within-season dynamics are coupled to recurrence equations that describe host and parasite between-season dynamics. The total population of new parasites at the end of the season, $v_2(T)$, gives rise to next season's starting parasite population, *i.e.* $v_1(0^+) = v_2(0^-) = \hat{v}(n)$. A parasite introduced into a naive host population persists or goes extinct depending on the length of the host emergence period and season length. For $\tau < T - t_l$, the stability of the parasite-free equilibrium is determined by the production of v_2 resulting from infection of s given by

$$\hat{v}(n+1) = v_2(T) = e^{-\delta(T - t_l - \tau)} (\frac{\alpha \beta e^{-\mu\tau} \hat{v}(n) \hat{s}(n)}{t_l} e^{-\delta t_l} \int_0^{t_l} e^{(-\mu u + \frac{\alpha \hat{v}(n) e^{-\delta u}}{\delta})} \int_0^u e^{(dx - \frac{\alpha \hat{v}(n) e^{-\delta x}}{\delta})} dx du + \\ \alpha \beta e^{-\mu\tau} \hat{v}(n) s(t_l) \int_0^{T - t_l - \tau} e^{-\frac{\alpha \hat{v}(n) e^{-\delta(u+t_l)}(-1+e^{\delta u})}{\delta} - \delta t_l - \mu u} du)$$

For $\tau > T - t_l$, the stability of the parasite-free equilibrium is determined by the production of v_2

resulting from infection of s given by

$$\hat{v}(n+1) = v_2(T) = \frac{\alpha\beta e^{-\mu\tau}\hat{v}(n)\hat{s}(n)}{t_l} e^{-\delta(T-\tau)} \int_0^{T-\tau} e^{(-\mu u + \frac{\alpha\hat{v}(n)e^{-\delta u}}{\delta})} \int_0^u e^{(\mu x - \frac{\alpha\hat{v}(n)e^{-\delta x}}{\delta})} dx du$$

The parasite-free equilibrium is unstable and a single parasite introduced into the system at the beginning of the season will persist if the density of v_2 produced by time T is greater than or equal to $\hat{v} = v_1(0) = 1$ (*i.e.* $v_2(T) \geq 1$, modulus is greater than unity). This expression is a measure of a parasite's fitness when rare given different host phenological patterns.

The total population of uninfected hosts at the end of the season, $s(T)$, reproduce and give rise to next season's host cohort, given by the map

$$\hat{s}(n+1) = \frac{\sigma s(T)}{1+\rho s(T)}$$

where σ is host fecundity and ρ is the density dependent parameter.

$$\hat{s}(n+1) = s(T) = \frac{\hat{s}(n)}{t_l} e^{(-\mu t_l + \frac{\alpha\hat{v}(n)e^{-\delta t_l}}{\delta})} e^{(-\mu(T-tl) - \frac{\alpha\hat{v}(n)e^{-\delta T}(1-e^{\delta(T-t_l)})}{\delta})} \int_0^{t_l} e^{(\mu u - \frac{\alpha\hat{v}(n)e^{-\delta u}}{\delta})} du$$

We can find the values of $\hat{v}*$ and $\hat{s}*$ numerically that satisfy

$$\begin{aligned} 1 =& e^{-\delta(T-t_l-\tau)} \Big(\frac{\alpha\beta e^{-\mu\tau}\hat{s}*}{t_l} e^{-\delta t_l} \int_0^{t_l} e^{(-\mu u + \frac{\alpha\hat{v}* e^{-\delta u}}{\delta})} \int_0^u e^{(\mu x - \frac{\alpha\hat{v}* e^{-\delta x}}{\delta})} dx du + \\ & \alpha\beta e^{-\mu\tau} s(t_l) \int_0^{T-t_l-\tau} e^{-\frac{\alpha\hat{v}* e^{-\delta(u+t_l)}(-1+e^{\delta u})}{\delta} - \delta t_l - du} du \Big) \\ 1 =& \frac{1}{t_l} e^{(-\mu t_l + \frac{\alpha\hat{v}* e^{-\delta t_l}}{\delta})} e^{(-\mu(T-tl) - \frac{\alpha\hat{v}* e^{-\delta T}(1-e^{\delta(T-t_l)})}{\delta})} \int_0^{t_l} e^{(\mu u - \frac{\alpha\hat{v}* e^{-\delta u}}{\delta})} du \end{aligned}$$

If we define $v(T) = x(\hat{v}*, \hat{s}*)$ and $s(T) = y(\hat{v}*, \hat{s}*)$, we can write the Jacobian for the between-season stability analysis as

$$\mathbf{J} = \begin{bmatrix} \frac{\partial x(\hat{v},\hat{s})}{\partial\hat{v}} & \frac{\partial x(\hat{v},\hat{s})}{\partial\hat{s}} \\ \frac{\partial y(\hat{v},\hat{s})}{\partial\hat{v}} & \frac{\partial y(\hat{v},\hat{s})}{\partial\hat{s}} \end{bmatrix}$$

We cannot solve this model further analytically as the partial derivatives w.r.t. $\hat{v}$ are transcendental. We are however able to find the eigenvalues of this Jacobian numerically. The leading eigenvalue is complex conjugate. The real part of the leading eigenvalue is less than one when the system dynamics are stable and greater than 1 when the system dynamics are cycling. This suggests that a Neimark-Sacker bifurcation drives the system to cycle (Strogatz 2018).

Appendix B

In Appendix B we find analytical solutions for equations 2a-e from the main text to study the evolution of parasite virulence given different host phenological patterns. Note that we primarily used numerical simulations in the main text to determine the outcome of parasite evolution as this analytical solution only holds when host and resident parasite populations are at a stable equilibrium when the mutant is introduced.

$$\frac{ds}{dt} = \hat{s}^* g(t, t_l) - \mu s(t) - \alpha s(t) v_1(t) - \alpha_m s(t) v_{1m}(t), \tag{B.1.a}$$
$$\frac{dv_{1m}}{dt} = -\delta_m v_{1m}(t), \tag{B.1.b}$$
$$\frac{dv_{2m}}{dt} = \alpha_m \beta_m e^{-\mu \tau_m} s(t - \tau_m) v_{1m}(t - \tau_m) - \delta_m v_{2m}(t). \tag{B.1.c}$$
$$\frac{dv_1}{dt} = -\delta v_1(t), \tag{B.1.d}$$
$$\frac{dv_2}{dt} = \alpha \beta e^{-\mu \tau} s(t - \tau) v_1(t - \tau) - \delta v_2(t), \tag{B.1.e}$$

with initial conditions: $s(0) = 0, v_{1m}(0^+) = v_{2m}(0^-), v_{2m}(\tau) = 0, v_1(0^+) = v_2(0^-), v_2(\tau) = 0$. m subscripts refer to the invading mutant parasite and its corresponding traits. $\hat{s}^*$ is the equilibrial host density determined in part by the resident parasite.

Again, separate solutions for (B.1.a-c) are required depending on whether τ and τ_m are greater or less than $T - t_l$. The length of τ relative to T and t_l determines $\hat{v}^*$ while the length of τ_m relative to

T and t_l determines the within-season dynamics of the mutant parasite. The solutions to all cases can be found in the code on Github (see "Code and data availability" in the main text for the link). We first show the solution to the case when $\tau_m < T - t_l$:

$$v_{1m}(t) = v_{1m}(0)e^{-\delta_m t} \qquad 0 < t < T$$

$$s(t) = \begin{cases} \frac{\hat{s}^*}{t_l}e^{(-\mu t+\frac{\alpha\hat{v}^* e^{-\delta t}}{\delta}+\frac{\alpha_m v_{1m}(0)e^{-\delta_m t}}{\delta_m})}\int_0^t e^{(\mu u-(\frac{\alpha\hat{v}^* e^{-\delta u}}{\delta}+\frac{\alpha_m v_{1m}(0)e^{-\delta_m u}}{\delta_m}))}du & 0 < t < t_l \\ s(t_l)e^{(-\mu(t-t_l)-(\frac{(\alpha\hat{v}^* e^{-\delta(t-t_l)}(-1+e^{\delta t})}{\delta}+\frac{\alpha_m v_{1m}(0)e^{-\delta_m(t-t_l)}(-1+e^{\delta_m t})}{\delta_m})} & t_l \leq t < T \end{cases}$$

$$v_{2m}(t) = \begin{cases} \frac{\alpha_m\beta_m e^{-\mu\tau_m}v_{1m}(0)\hat{s}^*}{t_l}e^{-\delta_m(t-\tau_m)}\int_0^{t-\tau_m}e^{(-\mu u+\frac{\alpha\hat{v}^* e^{-\delta u}}{\delta}+\frac{\alpha_m v_{1m}(0)e^{-\delta_m u}}{\delta_m})} \\ \int_0^u e^{(\mu x-(\frac{\alpha\hat{v}^* e^{-\delta x}}{\delta}+\frac{\alpha_m v_{1m}(0)e^{-\delta_m x}}{\delta_m}))}dxdu & \tau_m < t < t_l \\ e^{-\delta_m(t-t_l-\tau_m)}(v_2(t_l)+\alpha_m\beta_m e^{-\mu\tau_m}v_{1m}(0)s(t_l) \\ \int_0^{t-t_l-\tau_m}e^{-\frac{\alpha_m v_{1m}(0)e^{-\delta_m(u+t_l)}(-1+e^{\delta_m u})}{\delta_m}-\frac{\alpha\hat{v}^* e^{-\delta(u+t_l)}(-1+e^{\delta u})}{\delta}-\delta_m t_l-\mu u}du) & t_l \leq t \leq T \end{cases}$$

When $\tau_m > T - t_l$, the solution for $v_{2m}(t)$ is

$$v_{2m}(t) = \frac{\alpha_m\beta_m e^{-\mu\tau_m}v_{1m}(0)\hat{s}^*}{t_l}e^{-\delta_m(t-\tau_m)}\int_0^{t-\tau_m}e^{(-\mu u+\frac{\alpha\hat{v}^* e^{-\delta u}}{\delta}+\frac{\alpha_m v_{1m}(0)e^{-\delta_m u}}{\delta_m})}$$
$$\int_0^u e^{(\mu x-(\frac{\alpha\hat{v}^* e^{-\delta x}}{\delta}+\frac{\alpha_m v_{1m}(0)e^{-\delta_m x}}{\delta_m}))}dxdu \qquad \tau_m < t < T$$

The invasion fitness of a rare mutant parasite is given by the density of v_{2m} produced by the end of the season. When $\tau_m < T - t_l$, the mutant parasite invades in a given host phenological scenario if the density of v_{2m} produced by time T is greater than or equal to the initial $v_{1m}(0) = 1$ introduced at the start of the season ($v_{2m}(T) \geq 1$), following

$$v_{2m}(T) = e^{-\delta_m(T-t_l-\tau_m)}(v_2(t_l)+\alpha_m\beta_m e^{-\mu\tau_m}v_{1m}(0)s(t_l)$$
$$\int_0^{T-t_l-\tau_m}e^{-\frac{\alpha_m v_{1m}(0)e^{-\delta_m(u+t_l)}(-1+e^{-\delta_m u})}{\delta_m}-\frac{\alpha\hat{v}^* e^{-\delta(u+t_l)}(-1+e^{\delta u})}{\delta}-\delta_m t_l-\mu u}du)$$

When $\tau_m > T - t_l$, the mutant parasite invades in a given host phenological scenario if the density of v_{2m} produced by time T is greater than or equal to the initial $v_{1m}(0) = 1$ introduced at the start

of the season ($v_{2m}(T) \geq 1$), following

$$\begin{aligned} v_{2m}(T) =& \frac{\alpha_m \beta_m e^{-\mu \tau_m} v_{1m}(0) \hat{s}^*}{t_l} e^{-\delta_m (T-\tau_m)} \int_0^{T-\tau_m} e^{(-\mu u + \frac{\alpha \hat{v}^* e^{-\delta u}}{\delta} + \frac{\alpha_m v_{1m}(0) e^{-\delta_m u}}{\delta_m})} \\ & \int_0^u e^{(\mu x - (\frac{\alpha \hat{v}^* e^{-\delta x}}{\delta} + \frac{\alpha_m v_{1m}(0) e^{-\delta_m x}}{\delta_m}))} dx du \end{aligned}$$

We can use $v_{2m}(T)$ to find optimal virulence for a given host phenological scenario by finding the trait value that maximizes $v_{2m}(T)$. That is, the virulence trait, τ^*, that satisfies

$$\frac{dv_{2m}(T)}{d\tau_m}\Big|_{\tau_m=\tau_r} = 0 \tag{B.2}$$

$$\frac{d^2 v_{2m}(T)}{d\tau_m^2}\Big|_{\tau_m=\tau_r} < 0 \tag{B.3}$$

We primarily used numerical simulation to determine parasite evolutionary endpoints as the analytical solution does not reliably predict the invasion fitness of rare mutant parasites invading populations with cycling dynamics.

Literature citealtd

K. C. Abbott and G. Dwyer. Food limitation and insect outbreaks: complex dynamics in plant-herbivore models. Journal of Animal Ecology, pages 1004–1014, 2007.

R. M. Anderson and R. M. May. The population dynamics of microparasites and their invertebrate hosts. Philosophical Transactions of the Royal Society of London. B, Biological Sciences, 291 (1054):451–524, 1981.

W. Baltensweiler and A. Fischlin. The larch budmoth in the alps. In Dynamics of forest insect populations, pages 331–351. Springer, 1988.

I. Barber, B. W. Berkhout, and Z. Ismail. Thermal change and the dynamics of multi-host parasite life cycles in aquatic ecosystems. Integrative and comparative biology, 56(4):561–572, 2016.

A. Best. Host–pathogen coevolution in the presence of predators: fluctuating selection and ecological feedbacks. Proceedings of the Royal Society B, 285(1885):20180928, 2018.

S. Bewick, R. S. Cantrell, C. Cosner, and W. F. Fagan. How Resource Phenology Affects Consumer Population Dynamics. The American Naturalist, 187(2):151–166, Feb. 2016.

N. D. Burkett-Cadena, C. J. McClure, R. A. Ligon, S. P. Graham, C. Guyer, G. E. Hill, S. S. Ditchkoff, M. D. Eubanks, H. K. Hassan, and T. R. Unnasch. Host reproductive phenology drives seasonal patterns of host use in mosquitoes. PLoS One, 6(3):e17681, 2011.

R. W. Campbell. The gypsy moth and its natural enemies. Number 381. US Department of Agriculture, Forest Service, 1975.

T. Caraco and I.-N. Wang. Free-living pathogens: life-history constraints and strain competition. Journal of Theoretical Biology, 250(3):569–579, 2008.

J. F. Crow, M. Kimura, et al. An introduction to population genetics theory. An introduction to population genetics theory., 1970.

I. H. Crowell. The hosts, life history and control of the cedar-apple rust fungus gymnosporangium juniperi-virginianae schw. Journal of the Arnold Arboretum, 15(3):163–232, 1934.

V. Delucchi. Parasitoids and hyperparasitoids of zeiraphera diniana [lep., tortricidae] and their pole in population control in outbreak areas. Entomophaga, 27(1):77–92, 1982.

R. Donnelly, A. Best, A. White, and M. Boots. Seasonality selects for more acutely virulent parasites when virulence is density dependent. Proceedings of the Royal Society B: Biological Sciences, 280(1751):20122464, 2013.

G. Dwyer. Density dependence and spatial structure in the dynamics of insect pathogens. The American Naturalist, 143(4):533–562, 1994.

B. D. Elderd, J. Dushoff, and G. Dwyer. Host-pathogen interactions, insect outbreaks, and natural selection for disease resistance. The American Naturalist, 172(6):829–842, 2008.

R. Ferriere and M. Gatto. Chaotic population dynamics can result from natural selection. Proceedings of the Royal Society of London. Series B: Biological Sciences, 251(1330):33–38, 1993.

R. Ferriere and M. Gatto. Lyapunov exponents and the mathematics of invasion in oscillatory or chaotic populations. Theoretical Population Biology, 48(2):126–171, 1995.

C. Ferris and A. Best. The evolution of host defence to parasitism in fluctuating environments. Journal of theoretical biology, 440:58–65, 2018.

C. Ferris, R. Wright, M. A. Brockhurst, and A. Best. The evolution of host resistance and parasite infectivity is highest in seasonal resource environments that oscillate at intermediate amplitudes. Proceedings of the Royal Society B, 287(1927):20200787, 2020.

P. E. Fine and J. A. Clarkson. Measles in england and wales—i: an analysis of factors underlying seasonal patterns. International journal of epidemiology, 11(1):5–14, 1982.

B. F. Finkenstädt and B. T. Grenfell. Time series modelling of childhood diseases: a dynamical systems approach. Journal of the Royal Statistical Society: Series C (Applied Statistics), 49(2): 187–205, 2000.

E. Gaulin, C. Jacquet, A. Bottin, and B. Dumas. Root rot disease of legumes caused by aphanomyces euteiches. Molecular Plant Pathology, 8(5):539–548, 2007.

S. A. Geritz, G. Mesze, J. A. Metz, et al. Evolutionarily singular strategies and the adaptive growth and branching of the evolutionary tree. Evolutionary ecology, 12(1):35–57, 1998.

L. Govaert, E. A. Fronhofer, S. Lion, C. Eizaguirre, D. Bonte, M. Egas, A. P. Hendry, A. De Brito Martins, C. J. Melián, J. A. Raeymaekers, et al. Eco-evolutionary feedbacks—theoretical models and perspectives. Functional Ecology, 33(1):13–30, 2019.

J. Greenman, M. Kamo, and M. Boots. External forcing of ecological and epidemiological systems: a resonance approach. Physica D: Nonlinear Phenomena, 190(1-2):136–151, 2004.

K. Grunert, H. Holden, E. R. Jakobsen, and N. C. Stenseth. Evolutionarily stable strategies in stable

and periodically fluctuating populations: The rosenzweig–macarthur predator–prey model. Proceedings of the National Academy of Sciences, 118(4), 2021.

F. Hilker, T. Sun, L. Allen, and F. Hamelin. Separate seasons of infection and reproduction can lead to multi-year population cycles. Journal of theoretical biology, 489:110158, 2020.

J. L. Hite and C. E. Cressler. Resource-driven changes to host population stability alter the evolution of virulence and transmission. Philosophical Transactions of the Royal Society B: Biological Sciences, 373(1745):20170087, 2018.

J. Holuša and K. Lukášová. Pathogen's level and parasitism rate in ips typographus at high population densities: importance of time. Journal of Applied Entomology, 141(9):768–779, 2017.

D. W. Inouye and F. E. Wielgolaski. Phenology at high altitudes. In Phenology: An integrative environmental science, pages 249–272. Springer, 2013.

M. Kamo and A. Sasaki. The effect of cross-immunity and seasonal forcing in a multi-strain epidemic model. Physica D: Nonlinear Phenomena, 165(3-4):228–241, 2002.

M. J. Keeling and P. Rohani. Modeling infectious diseases in humans and animals. Princeton university press, 2011.

M. Kenis and J. Hilszczanski. Natural enemies of cerambycidae and buprestidae infesting living trees. In Bark and wood boring insects in living trees in Europe, a synthesis, pages 475–498. Springer, 2007.

D. A. Kennedy and G. Dwyer. Effects of multiple sources of genetic drift on pathogen variation within hosts. PLoS biology, 16(3):e2004444, 2018.

E. Kisdi. Evolutionary branching under asymmetric competition. Journal of Theoretical Biology, 197(2):149–162, 1999.

T. Klemola, M. Tanhuanpää, E. Korpimäki, and K. Ruohomäki. Specialist and generalist natural enemies as an explanation for geographical gradients in population cycles of northern herbivores. Oikos, 99(1):83–94, 2002.

J. C. Koella and M. Doebeli. Population dynamics and the evolution of virulence in epidemiological models with discrete host generations. Journal of Theoretical Biology, 198(3):461–475, 1999.

C. J. Krebs. Population fluctuations in rodents. University of Chicago Press, 2013.

S. Lion and J. A. Metz. Beyond r0 maximisation: on pathogen evolution and environmental dimensions. Trends in ecology & evolution, 33(6):458–473, 2018.

H. MacDonald, E. Akçay, and D. Brisson. Host phenology can drive the evolution of intermediate virulence strategies in some parasites. bioRxiv, 2021.

R. M. May. Regulation of populations with nonoverlapping generations by microparasites: a purely chaotic system. The American Naturalist, 125(4):573–584, 1985.

J. A. Metz, R. M. Nisbet, and S. A. Geritz. How should we define 'fitness' for general ecological scenarios? Trends in ecology & evolution, 7(6):198–202, 1992.

J. A. Metz, S. A. Geritz, G. Meszéna, F. J. Jacobs, and J. S. Van Heerwaarden. Adaptive dynamics: a geometrical study of the consequences of nearly faithful reproduction. 1995.

A. J. Miller-Rushing, T. T. Høye, D. W. Inouye, and E. Post. The effects of phenological mismatches on demography. Philosophical Transactions of the Royal Society B: Biological Sciences, 365(1555): 3177–3186, 2010.

J. H. Myers. Population cycles: generalities, exceptions and remaining mysteries. Proceedings of the Royal Society B: Biological Sciences, 285(1875):20172841, 2018.

J. H. Myers and J. S. Cory. Population cycles in forest lepidoptera revisited. Annual Review of Ecology, Evolution, and Systematics, 44:565–592, 2013.

S. H. Paull and P. T. Johnson. Experimental warming drives a seasonal shift in the timing of host-parasite dynamics with consequences for disease risk. Ecology letters, 17(4):445–453, 2014.

T. Schott, S. B. Hagen, R. A. Ims, and N. G. Yoccoz. Are population outbreaks in sub-arctic geometrids terminated by larval parasitoids? Journal of Animal Ecology, 79(3):701–708, 2010.

S. H. Strogatz. Nonlinear dynamics and chaos with student solutions manual: With applications to physics, biology, chemistry, and engineering. CRC press, 2018.

R. A. Taylor, A. White, and J. A. Sherratt. How do variations in seasonality affect population cycles? Proceedings of the Royal Society B: Biological Sciences, 280(1754):20122714, 2013.

P. Turchin. Complex population dynamics. Princeton university press, 2013.

M. van Asch and M. E. Visser. Phenology of forest caterpillars and their host trees: the importance of synchrony. Annu. Rev. Entomol., 52:37–55, 2007.

I.-N. Wang. Lysis timing and bacteriophage fitness. Genetics, 172(1):17–26, 2006.

A. White and R. G. Bowers. Adaptive dynamics of lotka–volterra systems with trade-offs: the role of interspecific parameter dependence in branching. Mathematical biosciences, 193(1):101–117, 2005.

A. White, J. Greenman, T. Benton, and M. Boots. Evolutionary behaviour in ecological systems with trade-offs and non-equilibrium population dynamics. Evolutionary Ecology Research, 8(3): 387–398, 2006.

N. J. White. Determinants of relapse periodicity in plasmodium vivax malaria. Malaria journal, 10 (1):1–36, 2011.

F. E. Wielgolaski and D. W. Inouye. Phenology at high latitudes. In Phenology: an integrative environmental science, pages 225–247. Springer, 2013.

M. Yamamichi, T. Yoshida, and A. Sasaki. Timing and propagule size of invasion determine its success by a time-varying threshold of demographic regime shift. Ecology, 95(8):2303–2315, 2014.

L. H. Yang and V. Rudolf. Phenology, ontogeny and the effects of climate change on the timing of species interactions. Ecology letters, 13(1):1–10, 2010.

E. I. Zehr et al. Control of brown rot in peach orchards. Plant disease, 66(12):1101–1105, 1982.
